# Embedded 3D Printing for Microchannel Fabrication in Epoxy-Based Microfluidic Devices

**DOI:** 10.3390/polym16233320

**Published:** 2024-11-27

**Authors:** Cheng Zhang, Wenyu Ning, Ding Nan, Jiangtao Hao, Weiliang Shi, Yang Yang, Fei Duan, Wenbo Jin, Lei Liu, Danyang Zhao

**Affiliations:** 1 State Key Laboratory of High-Performance Precision Manufacturing, School of Mechanical Engineering, Dalian University of Technology, Dalian 116024, China; 2 Zibo Vocational Institute, Zibo 255300, China; 3 National United Engineering Laboratory for Biomedical Material Modification, Dezhou 251100, China; 4 365th Research Institute, Northwestern Polytechnical University, Xi’an 710065, China

**Keywords:** embedded 3D printing, epoxy, fumed silica, microfluidic devices, yield-stress fluid

## Abstract

Microfluidic devices offer promising solutions for automating various biological and chemical procedures. Epoxy resin, known for its excellent mechanical properties, chemical resistance, and thermal stability, is widely used in high-performance microfluidic devices. However, the poor printability of epoxy has limited its application in 3D printing technologies for fabricating epoxy-based microfluidic devices. In this study, fumed silica is introduced into epoxy resin to formulate a yield-stress fluid suspension as a support bath for embedded 3D printing (e-3DP). The study demonstrates that increasing the fumed silica concentration from 3.0% to 9.0% (*w*/*v*) enhances the yield stress from 9.46 Pa to 56.41 Pa, the compressive modulus from 19.79 MPa to 36.34 MPa, and the fracture strength from 148.16 MPa to 168.78 MPa, while reducing the thixotropic time from 6.58 s to 1.32 s, albeit with a 61.3% decrease in the transparency ratio. The 6.0% (*w*/*v*) fumed silica–epoxy suspension is selected based on a balance between yield stress, transparency, and mechanical performance, enabling high-fidelity filament formation. Two representative microfluidic devices are successfully fabricated, demonstrating the feasibility of a fumed silica–epoxy suspension for the customizable e-3DP of epoxy-based microfluidic devices.

## 1. Introduction

Over the past few decades, microfluidic devices have attracted significant scholarly attention and have been widely adopted in fields such as medicine [1,2], chemistry [3,4], and biology [5,6]. Their advantages, including compact size, lightweight design, low power consumption, high sensitivity, and the potential for cost-effective mass production, make them ideal for the precise control, manipulation, and analysis of microscale fluids [7]. Recent advancements in 3D printing methods, such as inkjet printing (IJP) [8], selective laser sintering (SLS) [9], stereolithography (SLA) [10], two-photon polymerization (TPP) [11], and fused deposition modeling (FDM) [12], have enabled more efficient microfluidic device production, accelerating the path from design concepts to functional prototypes. Among these, epoxy resin-based microfluidic devices have been rapidly developed and widely applied in areas such as microchemical synthesis [13], chemical analysis [14], microvalves [15], cell culture [16], immunoassays [17], drug screening [18], and DNA analysis [19,20]. Epoxy resin is one of the most important thermosetting polymers, valued for its excellent biocompatibility, superior mechanical properties, chemical resistance, thermal stability, transparency, and toughness [2,21,22], which makes it a compelling choice for microfluidic device fabrication. However, the direct 3D printing of epoxy resin faces challenges, primarily due to its extended curing period and its inadequate yield-stress properties. As a result, methods such as laser ablation, soft lithography, and casting remain the dominant approaches for fabricating epoxy-based microfluidic devices [14,15,20]. Despite advancements in additive manufacturing, research on the direct 3D printing of customized epoxy-based microfluidic devices remains scarce.

Innovations in 3D printing techniques for epoxy materials are essential for expanding their use in the fabrication of microfluidic devices. In recent years, the support bath-assisted approach in 3D printing has attracted significant interest. This method allows for the creation of structures within a support bath exhibiting yield-stress and self-repair properties, with the medium being removed after printing. It facilitates the stable printing of complex structures using flexible materials like hydrogels and PDMS, ensuring the structural integrity of the printed designs. This technique is especially beneficial for liquid materials with prolonged crosslinking time, providing greater potential for fabricating high-precision, complex microfluidic devices. For instance, Arun et al. [22] used a Carbopol support bath to fabricate nonplanar 3D lattice structures with a high specific modulus using industrial-grade epoxy resin. Similarly, Weeks et al. [23] employed an epoxy-based ink to print periodic and random lattice structures within a granular silicone microgel support matrix. Although these studies did not focus on microchannel fabrication, they highlighted the potential for creating high-precision, complex architectures. However, two major limitations remain in the application of support bath-assisted 3D printing for microchannel fabrication. Firstly, when applying this technique, the entire microfluidic device is often printed rather than directly printing the embedded channels, resulting in lower manufacturing efficiency. Secondly, the layer-by-layer deposition process within the support bath leads to increased surface roughness due to the staircase effect, which can negatively impact cell culture performance [24].

In recent years, another support bath-assisted 3D printing technique, known as embedded 3D printing (e-3DP), has gained considerable attention for its potential to address the limitations of the aforementioned method. e-3DP enables the formation of hollow channels within curable support matrices through the removal of sacrificial inks, offering a robust method for fabricating smooth 2D or 3D microchannels. Currently, e-3DP has been applied in the fabrication of microfluidic devices. For instance, Hua et al. [25] used a fumed silica–PDMS suspension as the support bath and sacrificial ink for e-3DP, successfully fabricating two types of microfluidic chips for biomedical applications. Similarly, Alioglu et al. [26] used a silicone composite suspension as the support bath and xanthan gum as the sacrificial ink to successfully print complex microfluidic chips which were used to create stable microgels and vascular-like structures. However, PDMS microfluidic devices, unlike epoxy-based types, are unsuitable for experiments involving highly soluble solvents or reactive organic reagents. These conditions can cause detachment from glass substrates, dissolution of channel walls, and solvent-induced swelling, leading to geometric deformation of the channels. Furthermore, due to PDMS’s low elastic modulus, the PDMS microfluidic devices are prone to deformation under high-pressure conditions, compromising experimental reliability [14]. While epoxy microfluidic devices can overcome the aforementioned limitations, current e-3DP techniques are still not suitable for printing epoxy-based microfluidic devices. This is primarily because pure epoxy solutions do not possess adequate yield stress to support the printing of stable 2D or 3D structures. Additionally, epoxy exhibits hydrophobic properties [27], while commonly used sacrificial inks, such as gelatin and xanthan gum [26,28], are typically water-soluble. This disparity in properties generates pronounced interfacial tension between the epoxy suspension and the sacrificial ink filaments. Consequently, when the filament diameter falls below a critical threshold, it breaks under the influence of this tension, severely limiting the minimum achievable size of the printable channels [29,30]. Addressing these challenges requires tailoring the rheological properties of epoxy to facilitate e-3DP technology in fabricating microfluidic devices with intricate embedded channels.

In this study, fumed silica nanoparticles are dispersed into epoxy resin, forming a three-dimensional network that transforms the epoxy resin from a low-viscosity liquid into a yield-stress fluid. The rheology, mechanical properties, and transparency of the fumed silica–epoxy resin (FS–ER) suspensions are systematically examined across different silica concentrations to identify the optimal formulation for use as a support bath in the e-3DP microfluidic device. Following this, Pluronic F127 is chosen as a thermosensitive sacrificial ink due to its excellent amphiphilic properties. Small-diameter filaments are successfully printed within the FS–ER suspension, and their fidelity is evaluated. To validate this approach, two representative microfluidic devices are fabricated via e-3DP. In summary, this study integrates e-3DP technology with fumed silica–epoxy resin composites, proposing a new 3D printing method for epoxy-based microfluidic device fabrication and laying a technical foundation to address the poor printability of epoxy resin.

## 2. Materials and Methods

### 2.1. Material Preparation

The support bath was prepared by mixing an epoxy (ArtResin, Carrollton, TX, USA) base agent and hardener in a 1:1 volume ratio, as per the manufacturer’s guidelines. The mixture was stirred at 300 rpm for 2 min at room temperature using an overhead stirrer (Thermo Fisher Scientific, Waltham, MA, USA). Fumed silica powder (Aerosil^®^ R 812 S, Evonik, Parsippany, NJ, USA) was then added to the mixture at concentrations of 0.0%, 3.0%, 6.0%, and 9.0% (*w*/*v*). The fumed silica was dispersed by continuous stirring at 800 rpm for 5 min, ensuring even distribution of the particles throughout the epoxy resin. To remove any air bubbles that may have formed during mixing, the suspension was centrifuged at 2500 rpm for 5 min using a centrifuge (Cole-Parmer^®^ VS3400, Cole-Parmer Instrument Company, Vernon Hills, IL, USA). All preparation steps were performed at room temperature. A 6.0% (*w*/*v*) FS–ER suspension was specifically used for filament fidelity studies and as a support bath for printing two representative microfluidic devices.

To prepare the sacrificial ink, Pluronic F127 (P2443, Sigma-Aldrich, Burlington, MA, USA) was used as the ink for printing filaments within the FS–ER suspension. A 40.0% (*w*/*v*) Pluronic F127 solution was prepared by dispersing the appropriate amount of Pluronic F127 powder into deionized (DI) water at 4 °C. The solution was stirred at 800 rpm for 1 h to fully dissolve the Pluronic F127 powder. For printing filaments and microchannels, 0.1% (*v*/*v*) red food dye (McCORMICK & Co., Inc., Hunt Valley, MD, USA) was added to the Pluronic F127 solution to enhance its visibility. The mixture was stirred at 800 rpm for an additional 3 min to ensure that the dye was fully mixed with the Pluronic F127 solution. Following this, the solution was centrifuged at 2500 rpm for 3 min to remove any entrapped air bubbles. The Pluronic F127 solution was then aged by storing it at 4 °C for a minimum of 24 h. Before use, the sacrificial ink was brought to room temperature and left to stand for at least 2 h.

### 2.2. Rheological Property Characterization

A rheometer (MCR 92, Anton Paar, Ashland, VA, USA) with a cone-and-plate system was used to measure the rheological properties of the FS–ER suspensions with different fumed silica concentrations. The cone-and-plate system had a 1° cone angle, a 50.0 mm diameter, and a gap of 0.102 mm. Measurements were conducted at room temperature. To determine yield stress, steady shear rate sweeps were performed by increasing the shear rate from 0.01 to 1000 s^−1^, recording the corresponding shear stress (*τ*). The yield stress (*τ*_0_) of the material was calculated using the Herschel–Bulkley model: $\tau =\tau_{0}+k{\left(\dot{\gamma }\right)}^{n}$, where $\dot{\gamma }$ is the shear rate, *k* is the consistency index, and *n* is the flow index. Additionally, to evaluate the thixotropic time of the FS–ER suspensions at different silica concentrations, transient step shear rate sweeps were performed. The samples were pre-sheared at a shear rate of 10 s^−1^ for 20 s, and the shear rate was then instantaneously reduced to 0.1 s^−1^, and the viscosity change was recorded over the subsequent 200 s. To investigate the impact of the fumed silica concentration on the fluid-like behavior of the suspensions, frequency sweeps were performed. The strain was maintained at 1.0%, while the frequency was varied from 10^−1^ to 10^2^ Hz.

The rheological properties of the 40.0% (*w*/*v*) Pluronic F127 sacrificial ink were also measured using the rheometer. The thermosensitivity of the Pluronic F127 ink was characterized by performing temperature sweeps from 4 °C to 30 °C and back to 4 °C, with 1 °C increments, at a constant shear rate of 1 s^−1^, monitoring the viscosity changes. Additionally, steady shear rate sweeps were conducted at room temperature, with the shear rate increased from 10 s^−1^ to 1000 s^−1^ to examine the relationship between viscosity and shear rate. The zero shear rate viscosity of the sacrificial ink was then calculated based on these measurements. To investigate the fluid-like behavior of the ink, frequency sweeps were conducted at a controlled temperature of 25 °C with a strain of 1.0%, while varying the frequency from 10^−2^ to 10^1^ Hz.

### 2.3. Mechanical Property Measurement

To characterize the mechanical properties of the crosslinked FS–ER composites, uniaxial compression tests were performed on samples containing 0.0%, 3.0%, 6.0%, and 9.0% (*w*/*v*) concentrations of fumed silica. The uncured FS–ER suspensions were poured into custom cylindrical polytetrafluoroethylene (PTFE) molds (*Φ* 10.0 mm × 10.0 mm) and allowed to crosslink at room temperature for 12 h before demolding. After removal from the molds, the samples were left at room temperature for an additional 60 h, as per the manufacturer’s instructions, to ensure complete solidification of the FS–ER samples. The fully cured samples were mounted onto a universal mechanical testing machine (LE5105X, Lishi Instrument Co., Ltd., Shanghai, China) equipped with a 100 kN load cell. Testing was conducted under ambient conditions (room temperature: 25 °C) with a constant strain rate of 1.0 mm/min. The gauge length of each specimen was set to 10.0 mm. Force and displacement data were continuously recorded during the tests. Using the force-displacement data and the geometrical dimensions of the specimens, stress–strain curves were generated, from which the elastic modulus and fracture strength were determined for each sample.

### 2.4. Transparency Characterization

To evaluate the transparency of the crosslinked FS–ER composites at different fumed silica concentrations, a fixed volume of each suspension was poured into disposable acrylic Petri dishes (Acrylic jars, Beauticom Inc., Arcadia, CA, USA) and left to partially crosslink for 12 h. After demolding, the samples were allowed to fully cure for an additional 60 h. The final samples had a diameter of 35.0 mm and a thickness of 5.0 mm. For the transparency test, the samples were placed in transparent Petri dishes and positioned on an inverted fluorescence microscope (CKX53, Olympus, Beijing, China). A transmitted light source from the microscope was used to illuminate the samples, and images of the sample surfaces were captured from the opposite side of the sample. The optical intensity of the images was analyzed using ImageJ software (https://imagej.net/ij/ (accessed on 25 April 2024)). The transparency ratio was calculated by comparing the intensity of light transmitted through the FS–ER sample (*L_n_*) to the intensity of light transmitted through an empty Petri dish (*L*_0_), using the following formula: $\mathrm{transparency}\;\mathrm{ratio}=L_{n}/L_{0}$. Additionally, the clarity of the FS–ER samples was further evaluated by placing the samples over printed text. Images of the text, as seen through the samples, were captured using a digital camera (DC-FZ80, Panasonic, Osaka, Japan) to evaluate the sample’s visual transparency.

### 2.5. Printing System and Filament Printing Experiments

An e-3DP system, adapted from an FDM 3D printer (SOVOL 3D, Shenzhen Liandian Technology Co., Ltd., Shenzhen, China), was used to print epoxy filaments at varying extrusion pressures and path speeds. The printing was conducted at room temperature. A 6.0% (*w*/*v*) FS–ER suspension was used as the support bath for printing 40.0% (*w*/*v*) Pluronic F127 filaments. The printing conditions were as follows: printing pressures of 310 kPa (45 psi), 379 kPa (55 psi), and 448 kPa (65 psi) were applied, using a 23-gauge nozzle with an internal diameter of 340 μm and a length of 25.4 mm (EFD Nordson, Vilters, Switzerland). The path speeds were set to 0.4, 0.7, 1.0, 1.3, and 1.6 mm/s. During the printing process, the filament formation within the FS–ER suspension was recorded using a camera (120fps USB Camera, ELP, Shenzhen, China). Filament images were captured at 1, 3, 5, 30, 60, 300, 400, 500, 600, and 720 min after filament formation. ImageJ software was used to measure the dimensions of the filaments in the captured images to analyze the filament size changes before the complete crosslinking of the FS–ER suspension. A ruler was placed next to the printed filaments during both image and video capture as the reference scale. The scale was calibrated in ImageJ software, based on the known markings on the ruler to ensure accurate measurements.

### 2.6. Fabrication of Microfluidic Devices

To fabricate the microfluidic devices, sacrificial ink was deposited into a 6.0% (*w*/*v*) FS–ER suspension at room temperature using a 23-gauge nozzle to create designed patterns. For printing the cross-shaped channels, the printing pressure was set to 379 kPa, while 310 kPa was used for the ring-shaped channels, with a path speed maintained at 0.4 mm/s. After printing, the printed structures were left at room temperature for 12 h before demolding. The initially crosslinked FS–ER composites were then further cured at room temperature for an additional 60 h to ensure full crosslinking. Finally, the fully cured composites were refrigerated at 4 °C for 1 h to liquefy the sacrificial ink. The ink was then removed from the FS–ER composites, resulting in a microfluidic device with hollow channels. To test the functionality of the device, DI water dyed with black food dye (McCORMICK & Co., Inc., Hunt Valley, MD, USA) was manually injected into the channels. Additionally, the key dimensions of the channels were measured using ImageJ software to calculate the relative error of the printed channels.

### 2.7. Statistical Analysis

All measurements and experiments were repeated three times, and the quantitative values presented in the text and figures are reported as mean ± standard deviation (SD), with *n* = 3 samples per group.

## 3. Results

### 3.1. Mechanism of FS–ER Suspension-Assisted e-3DP

**Figure 1:**
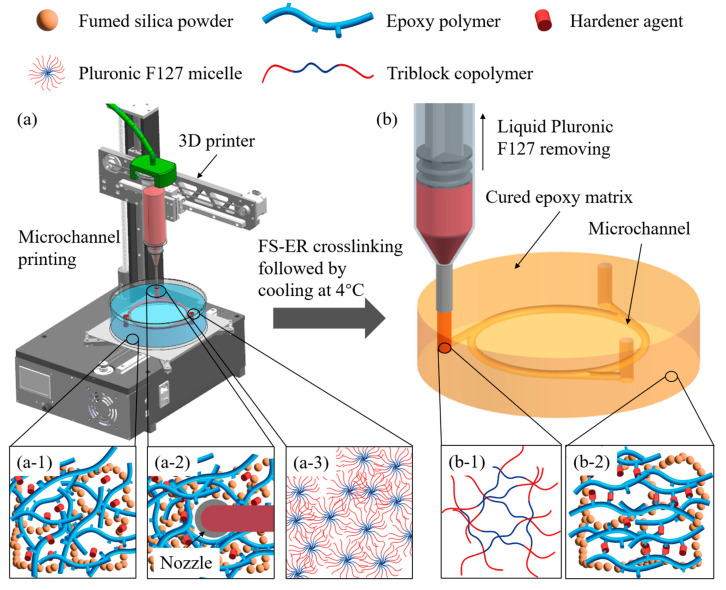
Schematic of the e-3DP mechanism assisted by FS–ER suspension for microfluidic device fabrication. (**a**) Printing of Pluronic F127 ink in FS–ER suspension: (**a-1**) three-dimensional network structure of fumed silica with freely moving epoxy polymer chains, (**a-2**) disordered fumed silica structure, under stress, with freely moving epoxy polymer chains, and (**a-3**) core-corona micelles structure of Pluronic F127. (**b**) Crosslinked FS–ER microfluidic device with hollow channels, formed after the removal of liquefied Pluronic F127: (**b-1**) freely moving triblock polymer chains of Pluronic F127 at 4 °C, and (**b-2**) interpenetrating network structure within the fully solidified FS–ER composite.

**Figure 2:**
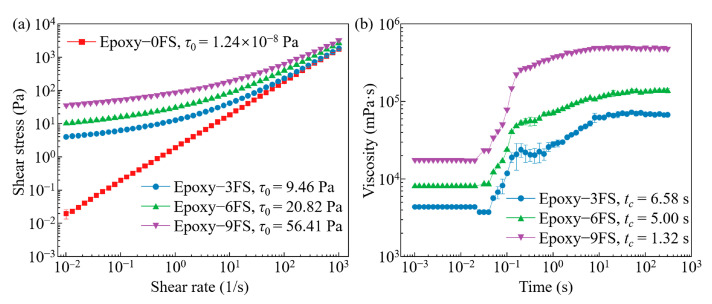
Rheological properties of FS–ER suspensions with different fumed silica concentrations: (**a**) shear stress as a function of shear rate, and (**b**) thixotropy tests on the FS–ER suspensions.

The method for printing microchannels in the FS–ER suspension is illustrated in Figure 1. The prepared FS–ER suspension consists primarily of an orderly arranged fumed silica network, with free, discrete epoxy molecular chains and hardener dispersed within the network, as shown in Figure 1(a-1). The basic units of fumed silica are microparticles with diameters ranging from 5 to 50 nm [31,32,33]. The surfaces of these nanoparticles contain functional groups, which interact with neighboring particles through intermolecular bonding, forming aggregates [34,35]. Without external forces, fumed silica particles aggregate into a robust 3D network, giving the suspension yield-stress properties and a solid-like behavior [36,37]. When an external force is applied, the bonds between fumed silica aggregates break down, leading to the fragmentation of the stable 3D structure and allowing the suspension to flow like a liquid, as depicted in Figure 1(a-2). This solid-to-liquid transition of the fumed silica suspension after yielding allows it to easily switch between solid and liquid states, making it a promising candidate as a support bath material for e-3DP applications [38].

**Figure 3:**
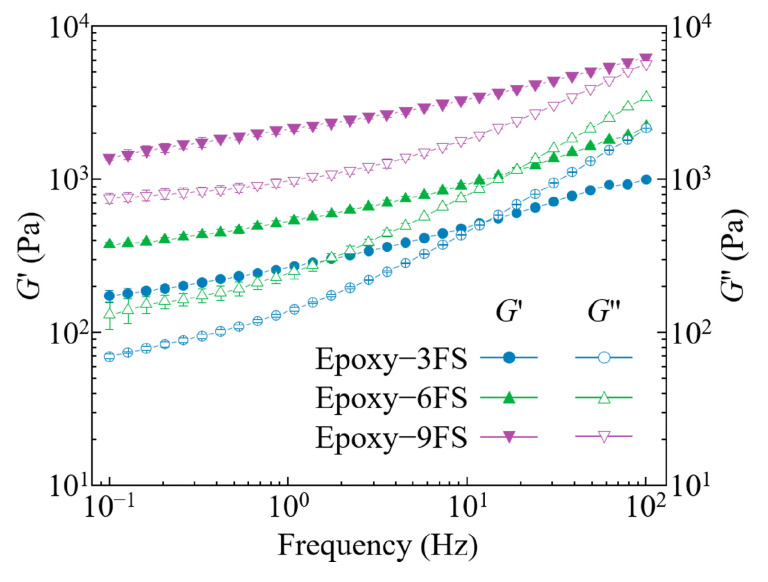
Shear moduli as a function of frequency for FS–ER suspensions.

During the printing process, the microstructure of the fumed silica enables the FS–ER suspension to provide in situ support for the extruded filaments. Pluronic F127, a triblock copolymer with excellent thermosensitive properties [39], is employed as the sacrificial ink in this study. At room temperature (25 °C), Pluronic F127 forms micelles with core-corona structures once its concentration surpasses a critical value, allowing it to transition into a gel-like state, as shown in Figure 1(a-3) [40,41]. As a result, Pluronic F127 transitions into a gel state after extrusion, enabling it to effectively maintain its structural integrity within the FS–ER composite. Within the FS–ER suspension, the epoxy undergoes initial crosslinking with the hardener over a 12 h period at room temperature, permitting subsequent demolding. After an additional 60 h of complete crosslinking, cooling the system to 4 °C induces the disassembly of the Pluronic F127 micelle structure, reverting it to its original copolymer chains and allowing for its safe removal without compromising the structural integrity (Figure 1(b-1)). By this stage, the epoxy has fully crosslinked with the hardener (Figure 1(b-2)), forming a stable three-dimensional network. The polymer chains are chemically bonded to the hardener through covalent bonds, resulting in an irreversible structure [42,43]. This method maintains the integrity of the microchannel structures, resulting in a fully formed microfluidic device, as per the intended design [38].

### 3.2. Characterization of Rheological Properties of FS–ER Suspensions

To evaluate the feasibility of using FS–ER suspensions as a support bath for e-3DP, a systematic study of their rheological properties is conducted. The yield-stress behavior of the FS–ER suspensions is evaluated using steady shear rate sweeps, the results of which are depicted in Figure 2. As the shear rate increases, a corresponding rise in shear stress is observed in the FS–ER suspensions across different fumed silica concentrations. At lower shear rates, suspensions with higher fumed silica concentrations (≥3.0% *w*/*v*) exhibit relatively high shear stress, indicating that the incorporation of fumed silica effectively imparts yield-stress behavior to the suspension.

To provide a more detailed quantification of this behavior, the shear stress and shear rate data are analyzed using the Herschel–Bulkley model [44,45], and the results indicate that pure epoxy exhibits negligible yield stress, confirming that the material itself lacks significant yield-stress properties. The yield-stress values for FS–ER suspensions with 3.0%, 6.0%, and 9.0% (*w*/*v*) fumed silica concentrations are calculated as 9.46, 20.82, and 56.41 Pa, respectively (Figure 2a). This demonstrates that the addition of fumed silica effectively transforms the epoxy solution from a simple viscous liquid into a yield-stress fluid, with yield stress increasing as the concentration of fumed silica rises. The yield-stress values for 3.0~9.0% (*w*/*v*) FS–ER suspensions are within the range typically required for support baths used in e-3DP [46].

To investigate the thixotropic time (*t_c_*) of the FS–ER suspensions and evaluate the efficiency of their transition between liquid and solid states, FS–ER suspensions with 3.0%, 6.0%, and 9.0% (*w*/*v*) fumed silica are subjected to high shear rates (10 s^−1^) in a steady shear state. This is followed by a rapid reduction in shear rate to 0.1 s^−1^. The viscosity change during this process is recorded, as shown in Figure 2b. Due to the shear-thinning effect, the microstructure of the fumed silica nanoparticles is disrupted, resulting in relatively low viscosity during the pre-shear phase. However, upon the significant reduction in shear rate, the viscosity of the FS–ER suspensions rapidly increases within approximately 0.2 s, indicating the quick recovery of the fumed silica nanoparticles into a solid-like three-dimensional network. The thixotropic time (*t_c_*) is defined as the time required for the suspension to recover to 80% of its stable viscosity at a shear rate of 0.1 s^−1^ [47,48]. The measured thixotropic times (*t_c_*) for FS–ER suspensions containing 3.0%, 6.0%, and 9.0% (*w*/*v*) fumed silica are 6.58, 5.00, and 1.32 s, respectively. This indicates that the thixotropic time decreases as the fumed silica concentration increases. For e-3DP, a shorter thixotropic time is desirable so that the support bath material can quickly transition from a liquid to a solid-like state, filling the voids left by the moving nozzle and supporting the deposited structure in situ [30].

The results of the frequency sweeps are illustrated in Figure 3. At a high fumed silica concentration (9% (*w*/*v*) FS), *G*′ exceeds *G*′′ across the frequency sweep range, indicating that the incorporation of high concentrations of fumed silica transforms the epoxy into a solid-like state. As the fumed silica concentration decreases, both *G*′ and *G*′′ decline, with *G*′ reducing more rapidly. This results in the lower concentration FS–ER (3% (*w*/*v*) FS) behaving as a solid-like material at low frequencies but shifting towards liquid-like behavior at higher frequencies. To ensure the stability of the printed filaments, support bath materials with predominantly solid-like behavior should be selected to effectively fix the filaments in their deposited positions.

### 3.3. Characterization of Mechanical Properties of FS–ER Composites

**Figure 4:**
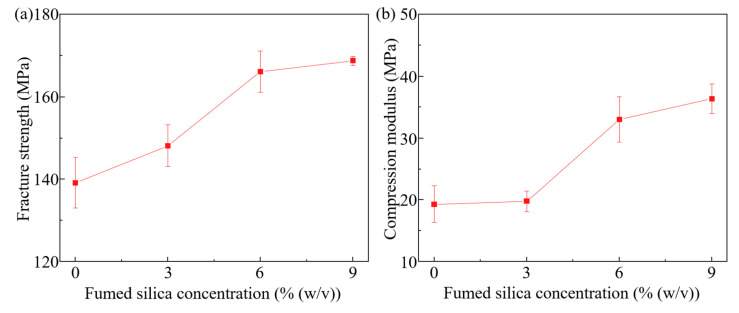
Mechanical properties of crosslinked FS–ER samples: (**a**) fracture strength as a function of fumed silica concentration and (**b**) compression modulus as a function of fumed silica concentration.

Compression tests are conducted on the FS–ER composites, as microfluidic devices are typically subjected to compressive stresses during various applications [49]. Exploring the compressive mechanical properties of the material is therefore crucial for its utilization in microfluidic device fabrication. The impact of fumed silica concentration on the mechanical properties of crosslinked FS–ER composites is systematically investigated, with the results presented in Figure 4. The results demonstrate a consistent increase in both the elastic modulus and fracture strength as the fumed silica concentration increases. Specifically, when 3.0% (*w*/*v*) fumed silica is incorporated, the compressive modulus and fracture strength of the FS–ER composites are measured at 19.79 MPa and 148.16 MPa, respectively, marginally surpassing those of pure epoxy, which exhibits a compressive modulus of 19.31 MPa and a fracture strength of 139.17 MPa. In contrast, at a fumed silica concentration of 9.0% (*w*/*v*), the compressive modulus and fracture strength surge to 36.34 MPa and 168.78 MPa, highlighting the substantial mechanical reinforcement imparted by the silica addition.

The enhanced mechanical properties of the epoxy can be attributed to the formation of an interpenetrating organic–inorganic network between the fumed silica and the epoxy resin [50]. The addition of silica leads to the development of an inorganic network within the epoxy resin, where the epoxy polymer chains penetrate and interweave with the silica inorganic network structure, forming an interpenetrating organic–inorganic structure [51]. Furthermore, strong interfacial interactions and adhesion between the epoxy matrix and the silica network, enhanced by the aerogel’s high surface area and pore volume, significantly improve the composites’ mechanical properties [50,52]. According to existing research, cellular behaviors such as growth and proliferation are greatly influenced by the mechanical characteristics of the substrate [53]. By adjusting the concentration of fumed silica, these mechanical properties can be modulated, facilitating the fabrication of microfluidic devices with customizable characteristics.

### 3.4. Characterization of Transparency of FS–ER Suspensions

**Figure 5:**
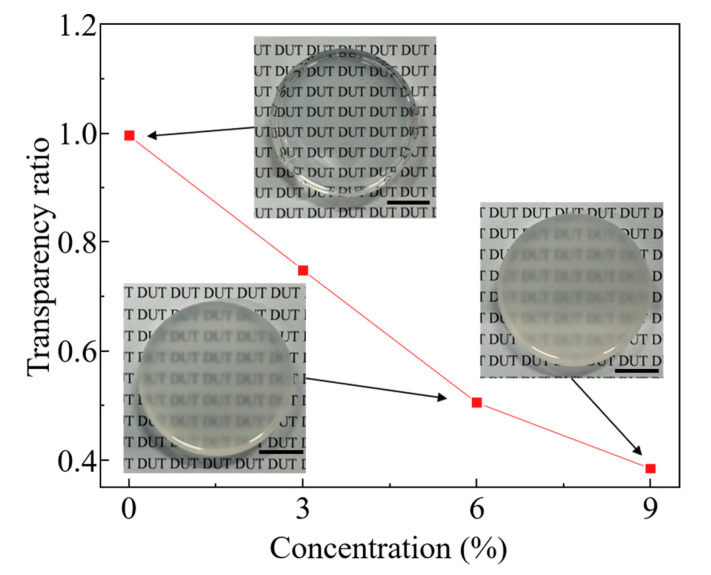
Transparency of crosslinked FS–ER samples with different fumed silica concentrations. Scale bar: 10 mm.

Microfluidic devices are commonly used for cell culture, bioreactor processes, and drug screening experiments, and other applications in which researchers need to observe fluid flow, cell behavior, and reaction processes inside the device in real time. Therefore, microfluidic devices must have good transparency to facilitate accurate quantitative analysis. To characterize the transparency of the crosslinked FS–ER samples, the transparency ratio of the cast FS–ER samples is tested using an inverted fluorescence microscope. This method evaluates the light transmittance of the resin sheets by comparing the intensity of light passing through the samples under the same light source. Similar approaches are commonly employed for assessing the haze and transmittance of materials [25,54]. Figure 5 shows the results for samples with different fumed silica concentrations (0.0, 3.0, 6.0, and 9.0% (*w*/*v*)). It is observed that the transparency ratio of the pure epoxy sample (0.0% (*w*/*v*) fumed silica) was 0.996, close to 1.0, indicating almost no obstruction of light transmission. However, a progressive reduction in transparency is observed as the fumed silica concentration increases. At a concentration of 9.0% (*w*/*v*), the transparency ratio decreases to 0.385, representing a 61.3% reduction compared to that of pure epoxy, and the text beneath the cast FS–ER sample is no longer clearly visible. The decrease in transparency is attributed to the formation of a dense three-dimensional silica network, which leads to enhanced light scattering and refraction through the material. With increasing fumed silica concentration, this scattering effect becomes more pronounced, leading to further reductions in transparency [55,56]. Therefore, considering the combined effects of the fumed silica concentration on the rheological properties, mechanical performance, and transparency of FS–ER composites, 6.0% (*w*/*v*) FS–ER is selected as the support bath material for the e-3DP of high mechanical strength microfluidic devices.

### 3.5. Filament Formation in FS–ER Suspension

Although the 6.0% (*w*/*v*) FS–ER composite exhibits excellent mechanical strength, transparency, and suitable rheological properties, its suitability for the embedded printing of microchannels in microfluidic devices remains to be demonstrated. To validate the hypothesis that FS–ER suspensions support stable filament formation, we have explored the mechanism of sacrificial ink filament formation within the 6.0% (*w*/*v*) FS–ER suspension. Epoxy, as a high-performance thermosetting polymer, is widely used in microfluidic devices due to its excellent mechanical strength, chemical resistance, and thermal stability [57]. However, pure epoxy in its liquid state exhibits low viscosity, lacks yield-stress properties, and its curing process is relatively slow (taking hours to days, depending on the formulation and curing temperature). This makes it difficult to directly fabricate using conventional 3D printing methods, typically requiring other methods such as casting [15]. Therefore, in this study, fumed silica is incorporated into the epoxy system to improve its rheological properties and mechanical performance after crosslinking. Additionally, we evaluate its effectiveness as a support bath for e-3DP. Specifically, we investigate the filament formation process and the evolution of filament diameter over time by depositing 40.0% (*w*/*v*) Pluronic F127 ink into the 6.0% (*w*/*v*) FS–ER suspension.

**Figure 6:**
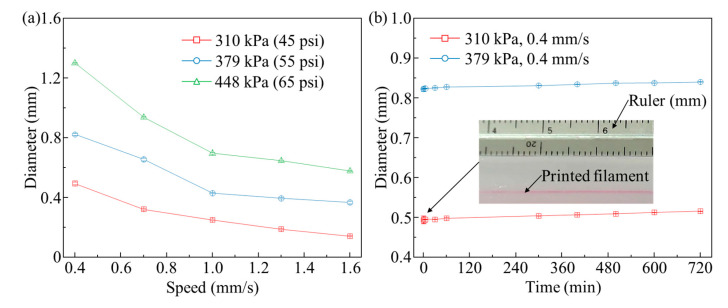
Filament formation in FS–ER suspension: (**a**) filament diameter as a function of time at different path speeds and extrusion pressures, and (**b**) filament diameter as a function of resting time after printing.

First, the filament diameters under different extrusion pressures and path speeds are measured using ImageJ software. This method is commonly used in studies involving embedded printing with support baths for the dimensional analysis of printed structures [58,59]. As shown in Figure 6a, the results indicate that the filament diameter increases with a higher extrusion pressure, while higher path speeds lead to reduced filament diameters. At a speed of 0.4 mm/s, when the pressure increases from 310 kPa to 448 kPa, the filament diameter increases from approximately 0.49 mm to 1.30 mm. Conversely, at a pressure of 310 kPa, when the path speed increases from 0.4 mm/s to 1.6 mm/s, the filament diameter significantly decreases from around 0.49 mm to 0.14 mm. When performing e-3DP in a yield-stress support bath, the relationship between filament diameter (*D*), extrusion pressure (*p*_0_), and printing path speed (*v_p_*) can be described by the following equation: $D=\sqrt{\left[\left(p_{0}-\sigma_{0}\right)+\rho gL\right]R^{4}/2\eta_{0}Lv_{p}}$, where *σ*_0_ represents the yield stress of the support bath, *ρ* and *η*_0_ are the density and zero shear viscosity of the ink material, *g* is the gravitational acceleration, and *L* and *R* are the length and radius of the dispensing nozzle, respectively [60]. Therefore, at a constant extrusion pressure, the filament diameter decreases with increasing path speed, while at constant path speed, a higher extrusion pressure results in larger filament diameters.

To investigate the variation in filament diameter before the FS–ER suspension fully crosslinks, filaments are printed in the support bath at an extrusion pressure of 310 kPa and a speed of 0.4 mm/s, as well as at 379 kPa and 0.4 mm/s. The diameter changes of the filaments are recorded over a 12 h period. As shown in Figure 6b, over a 12 h observation period, no significant changes in filament diameter are detected post-printing. This stability is attributed to the strong interfacial tension between the hydrophobic FS–ER suspension [61] and the hydrophilic poly(ethylene oxide) (PEO) component of Pluronic F127 [39], which creates an effective barrier that prevents the diffusion of Pluronic F127 into the epoxy suspension. As a result, this interfacial tension plays a role in stabilizing the filament structure, leading to minimal changes in filament diameter over time within the suspension.

In e-3DP, the formation of filaments is governed by a combination of factors, including the properties of both the support bath and ink materials, as well as the printing parameters. For the support bath material, a short thixotropic time and high yield stress are desirable [38]. First, the thixotropic time refers to the time required for the support bath material to transition from a liquid to a solid state once stress is removed. During the printing process, if the transition of the bath material from liquid to solid is too slow, the support bath may fail to capture and stabilize the deposited filament immediately after extrusion, leading to irregular filament formation [62]. In contrast, a shorter thixotropic time can facilitate more effective ink deposition and better retention of structural features [48]. Therefore, in FS–ER suspension-assisted e-3DP, minimizing the thixotropic time is crucial [63]. Yield stress, which is the applied stress threshold that causes the support bath to transition from a solid to a liquid state, is another crucial factor in the design of support bath materials. When the yield stress is insufficient, the support bath may fail to stably capture the printed filament and secure it in place, thus compromising the structural integrity of the printed object [58]. In this study, after considering the yield stress, thixotropic time, and transparency of the materials, the 6.0% (*w*/*v*) FS–ER suspension is selected as the support bath for e-3DP.

**Figure 7:**
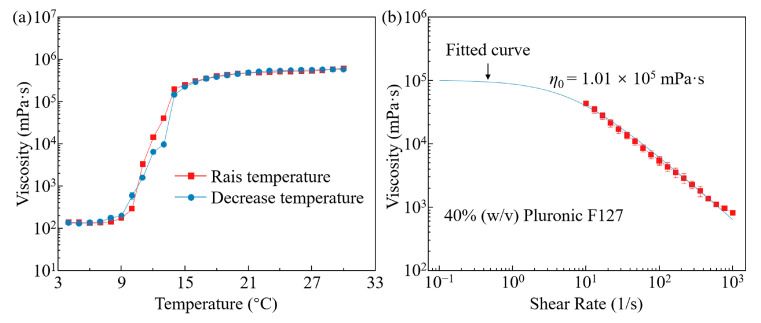
Rheological properties of 40.0% (*w*/*v*) Pluronic F127: (**a**) viscosity as a function of temperature and (**b**) viscosity as a function of shear rate.

In addition to the rheological properties of the support bath, the rheological characteristics of the ink itself are also key factors in determining the morphology and size of the extruded filaments. The viscosity (*η*) of 40.0% (*w*/*v*) Pluronic F127 is measured under different temperatures, along with its viscosity as a function of shear rate ($\dot{\gamma }$) at room temperature. As shown in Figure 7a, during the heating stage, when the temperature reaches approximately 6 °C, the viscosity of Pluronic F127 begins to increase from around 1.4 × 10^2^ mPa·s due to micelle nucleation. This is followed by a sharp increase in viscosity as micelle growth transforms the homogeneous fluid into a biphasic system. Finally, the viscosity stabilizes at approximately 5.1 × 10^5^ mPa·s at around 21 °C, marking the completion of the gelation process. The viscosity–temperature curve during the cooling phase is almost identical to that of the heating phase. As a result, the sacrificial ink remains in a gel state at room temperature and fully liquefies at 4 °C during both the heating and cooling cycles.

Additionally, the viscosity–shear rate data for 40.0% (*w*/*v*) Pluronic F127 are presented in Figure 7b. By fitting the data to the Carreau-like model [64] $\eta \left(\dot{\gamma }\right)=\eta_{0}/\left(1+{\left(k\dot{\gamma }\right)}^{n}\right)$, where *k* is the consistency index and *n* is the flow index, the zero shear viscosity (*η_0_*) of the material is determined to be 1.01 × 10^5^ mPa·s. Based on the formula $v_{out}=p_{0}R^{2}/8\eta_{0}L$ [60], with an extrusion pressure (*p*_0_) of 310 kPa, a nozzle radius (*R*) of 0.17 mm, and a nozzle length (*L*) of 25.4 mm, the ink extrusion speed (*v_out_*) is calculated to be approximately 0.45 mm/s. When the path speed is lower than *v_out_*, excess deposition of ink during printing leads to a filament diameter larger than the nozzle diameter, resulting in an expanded filament. Conversely, as the path speed surpasses *v_out_*, the movement of the nozzle stretches the deposited filaments, creating a filament with a diameter smaller than the nozzle diameter [58]. This is consistent with the experimental results. As shown in Figure 6a, at an extrusion pressure of 310 kPa, when the speed is lower than *v_out_*, such as at 0.4 mm/s, the filament diameter is 0.49 mm, exceeding the nozzle diameter (0.34 mm), indicating filament swelling. When the path speed exceeds *v_out_*, such as at 0.7 mm/s, the filament diameter is 0.32 mm, smaller than the nozzle diameter, indicating a stretched filament. Stretched filaments are prone to shrinkage when the support bath yield stress is insufficient, making it difficult to maintain filament stability [58]. Additionally, higher path speeds of the nozzle may interfere with previously printed filaments, potentially damaging the already printed structures [26,65]. Therefore, this study selects a 310 kPa extrusion pressure with a path speed of 0.4 mm/s and a 379 kPa extrusion pressure with a path speed of 0.4 mm/s as the printing parameters for subsequent microchannel printing.

Based on the selected printing parameters, the extrusion shear rate of the ink (${\dot{\gamma }}_{ink}$) and the shear rate in the support bath (${\dot{\gamma }}_{sup}$) at the nozzle tip during printing can be estimated using the equations ${\dot{\gamma }}_{ink}=v_{out}/d_{i}$ and ${\dot{\gamma }}_{sup}=v_{p}/d_{o}$, where *d_i_* is the nozzle inner diameter and *d_o_* is the nozzle outer diameter (the outer diameter of a 23G nozzle is 0.64 mm) [66]. For the support bath, when the nozzle moves at 0.4 mm/s, the shear rate is calculated to be 0.63 s^−1^. As shown in Figure 2a, the corresponding shear stress on the support bath at this shear rate is approximately 26 Pa, which exceeds the yield stress of the material. This indicates that the support bath around the nozzle is fully liquefied, facilitating crack healing behind the nozzle. For the ink material, under an extrusion pressure of 310 kPa, *v_out_* is calculated using the above formula, resulting in a value of 0.45 mm/s. The shear rate is thus 1.32 s^−1^. According to Figure 7b, the viscosity of the ink under these conditions is approximately 8.38 × 10^4^ mPa·s. At an extrusion pressure of 379 kPa, *v_out_* increases to 0.53 mm/s, and the shear rate rises to 1.56 s^−1^. The ink viscosity under these conditions is approximately 8.12 × 10^4^ mPa·s. The high viscosity of the ink during extrusion under both pressures reduces its diffusion into the support bath, enhances the fidelity of the printed structures, ensuring the stability of the printed filaments [58].

**Figure 8:**
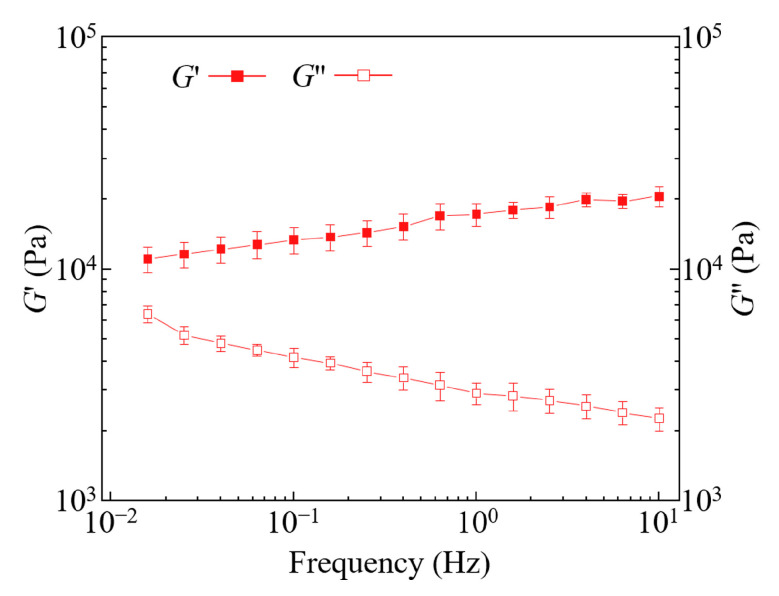
Shear moduli as a function of frequency for 40% *w*/*v* Pluronic F127.

The linear viscoelastic properties of 40% Pluronic F127 gels are investigated through frequency sweep experiments, and the results are presented in Figure 8. Across the tested frequency range, the storage modulus (G′) exceeds the loss modulus (G′′), indicating that the sample exhibits the viscoelastic solid-like behavior characteristic of gels. This confirms that the ink remains in a solid state after deposition into the support bath, which is beneficial for maintaining filament stability.

### 3.6. Fabrication of Representative Microfluidic Devices

**Figure 9:**
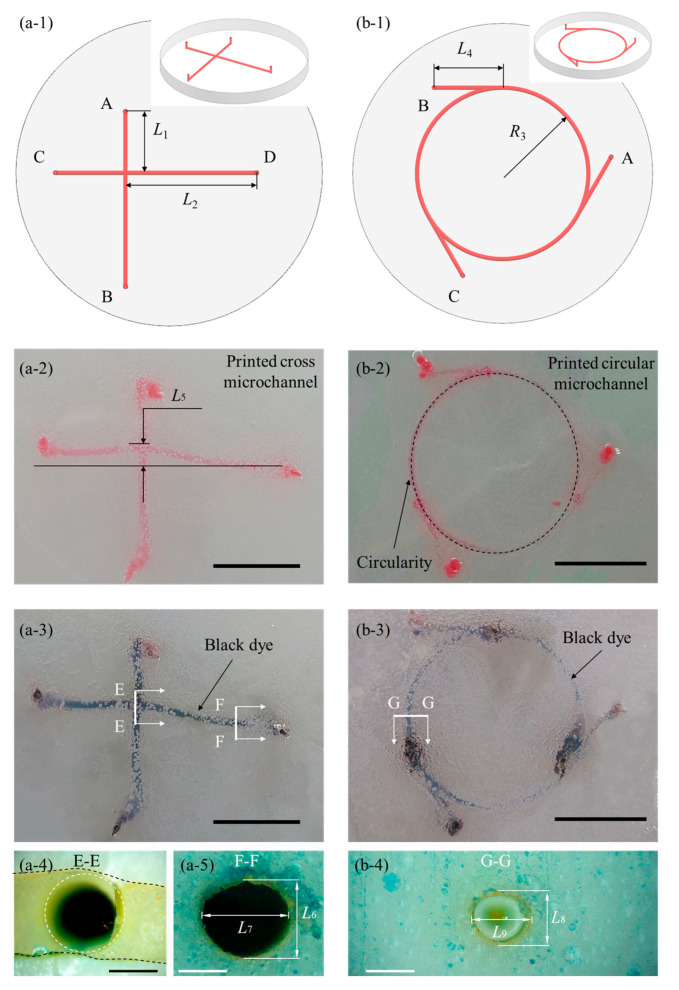
Printed microfluidic devices: (**a-1**) 3D model of the designed cross-channel microfluidic device, (**a-2**) printed cross-channel microstructure, and (**a-3**) cross-channel microfluidic device infused with black dye after the removal of the sacrificial ink. (**a-4**,**a-5**) Cross-sectional views of the microchannels at corresponding positions in the cross-channel microfluidic device. (**b-1**) A 3D model of the designed circular-channel microfluidic device, (**b-2**) printed circular-channel microstructure, and (**b-3**) circular-channel microfluidic device infused with black dye after the removal of the sacrificial ink. (**b-4**) Cross-sectional view of the microchannel in the circular-channel microfluidic device. The scale bars in (**a-2**,**a-3**,**b-2**,**b-3**) represent 10 mm, while the scale bars in (**a-4**,**a-5**,**b-4**) represent 0.5 mm.

Based on the above printing parameters and filament formation studies, complex 3D structures are successfully printed within the 6.0% (*w*/*v*) FS–ER suspension, resulting in the fabrication of epoxy-based microfluidic devices with embedded microchannels. Drawing from existing research on microfluidic devices, we design two representative microchannel structures: a cross-shaped channel and a circular channel, as illustrated in Figure 9(a-1,b-1). The cross-shaped channel, composed of two orthogonal channels (Figure 9(a-1)), has been previously used for the separation and detection of dopamine and catechol [67]. Microchannels in microfluidic devices typically have diameters ranging from 100 to 1000 μm [8], and in this study, the cross-channel diameter is set to 800 μm, and based on this, the extrusion pressure and path speed are chosen as 379 kPa and 0.4 mm/s. To improve the visibility of the printed filaments, red dye is added to the sacrificial ink. The fully crosslinked epoxy-based microfluidic device is shown in Figure 9(a-2). The results demonstrate that the FS–ER suspension can effectively maintain the deposited filaments in place. Subsequently, the crosslinked FS–ER composite is stored at 4 °C to liquefy and remove the sacrificial Pluronic F127 ink. After removal, a cross-shaped microfluidic device with hollow channels is obtained. Finally, black-dyed DI water is injected into the channels from the A and B outlets (Figure 9(a-1)) to test the channel continuity. As shown in Figure 9(a-3), all channels are filled with the black dye, confirming the continuity of the microchannels through the device.

Subsequently, a circular microfluidic device with a channel diameter of 500 μm is designed, as shown in Figure 9(b-1). This microchannel structure has been previously reported for use in biochemical reactions such as the polymerase chain reaction (PCR) [68]. The extrusion pressure and path speed for the e-3DP of this microfluidic device are set to 310 kPa and 0.4 mm/s, respectively. The printed circular microchannels exhibit well-defined boundaries, as shown in Figure 9(b-2). The flow continuity of the channels is demonstrated by injecting DI water mixed with black dye through inlet A of the device, as illustrated in Figure 9(b-3).

The key dimensions of the printed microfluidic devices, as shown in Figure 9(a-1,b-1), are measured, and the results are summarized in Table 1. As shown in the table, the relative errors in the key dimensions are all less than 0.88%. In Figure 9(a-1), the horizontal and vertical channels are labeled as CD and AB, respectively. The printing sequence starts with the horizontal CD channel, followed by the vertical AB channel, which is printed from point B to point A. Consequently, as depicted in Figure 9(a-2), the AB channel maintains good linearity, while the CD channel exhibits bending deformation due to disturbances caused by the moving nozzle. Using ImageJ software, we measure the deflection (*L*_5_) of the CD channel, as shown in Figure 9(a-2). The measured *L*_5_ = 1.87 ± 0.05 mm. The deflection ratio (*α*) is calculated using the formula $\alpha =L_{5}/designed\;length\;of\;CD$, resulting in *α* = 8.13%. Additionally, as shown in Figure 9(b-2), the circularity (*β*) of the printed circular channel is evaluated using ImageJ software based on the formula $Circularity=4\pi \times Area/Perimeter^{2}$. A *β* value closer to 1 indicates a shape closer to a perfect circle. The circularity of the printed circular channel is calculated as *β* = 0.88 ± 0.02. The observed distortion in the shape of the printed filaments is primarily attributed to the liquefied region generated by the movement of the nozzle during the printing process. This phenomenon is inherently determined by the material properties of the yield-stress support bath, which liquefies under applied stress. According to existing studies [65], reducing the nozzle diameter, decreasing the printing speed, and increasing the yield stress of the support bath material are effective strategies to minimize the disturbances to the previously printed structures caused by nozzle movement. In future research, we plan to further optimize the printing parameters and refine the formulation of the support bath to enhance the printing precision of microfluidic devices.

The cross-sections of the microchannels at the locations shown in Figure 9(a-3,b-3) are presented in Figure 9(a-4), 9(a-5), and 9(b-4), respectively. As shown in Figure 9(a-4), the orthogonal channels are well-fused, ensuring the functionality of the microfluidic device. Figure 9(a-5,b-4) reveals that the cross-sectional shapes of the microchannels are approximately elliptical. Using ImageJ software, the characteristic dimensions of the microchannel cross-sections are measured, and the results are summarized in Table 2. As indicated in Table 2, the height of the microchannels closely matches the designed values, while their width is larger than expected. This discrepancy is likely due to the resistance exerted by the support bath on the filament as it is extruded, causing the filament to expand laterally and form an elliptical cross-section. By further optimizing the rheological properties of the ink and support bath materials, it is expected that more circular cross-sectional microchannels can be achieved in future printing [66].

Despite some dimensional deviations between the printed microchannels and their designed specifications, the continuity of the microchannels has been successfully verified, demonstrating the functionality and usability of the printed microfluidic devices. From these findings, it can be concluded that e-3DP technology enables the efficient fabrication of epoxy-based microfluidic devices. Compared with traditional microfabrication methods for microfluidic devices, such as photolithography, micromachining, micro-milling, hot embossing, and injection molding, e-3DP simplifies the manufacturing process, reduces the number of steps and time required, and minimizes the dependence on costly equipment such as cleanrooms. This makes microfluidic device fabrication in a laboratory environment more convenient and flexible. Furthermore, 3D printing offers design flexibility, allowing for the rapid customization of different microfluidic structures as needed, which could further expand the application scope for microfluidic devices.

## 4. Conclusions and Future Work

This work verifies the feasibility of using FS–ER suspensions as a support medium in e-3DP to fabricate high-strength epoxy-based microfluidic devices. It is observed that higher concentrations of fumed silica notably improve the rheological behavior and mechanical strength, though transparency decreases as a trade-off. The printing parameters significantly influence filament formation, with higher path speeds and lower extrusion pressures resulting in smaller filament diameters. Filaments maintain their stability in the FS–ER suspension throughout the epoxy crosslinking process, enabling the successful fabrication of two microfluidic devices with intact channel structures.

Despite the merits of this approach, including its flexibility, low cost, and ease of fabrication, several challenges remain to be addressed in future work. For example, achieving higher shape fidelity in microfluidic devices and smaller, more circular cross-sectional channels will require further optimization of the epoxy-based support bath formulation and the associated printing conditions. Additionally, future work will involve mechanical testing, including three-point bending, internal pressure, and tensile strength tests, to comprehensively evaluate the impact of the embedded network on the device’s strength, flexibility, and interlayer adhesion. The current FS–ER suspension presents a trade-off between rheological performance, mechanical strength, and transparency, suggesting a need for the development of novel yield-stress additives that can maintain the suspension’s transparency while enhancing its yield-stress behavior. Moreover, this study primarily focuses on 2D microchannel fabrication. Future research could delve into the e-3DP of 3D microchannel systems, requiring innovations in path planning and minimizing disruptions from nozzle motion. In addition, expanding the range of functional applications, especially within the biomedical field, is a promising direction. Future efforts will target the creation of microfluidic systems with intricate 3D channels and compartments, tailored for applications such as cell culture, organ-on-a-chip systems, and high-throughput drug assays. These improvements could significantly extend the utility of microfluidic devices in chemical and regenerative medicine contexts.

## Figures and Tables

**Table 1:** Key dimensions of the microfluidic devices.

Key Dimension	Design Value (mm)	Measured Value (mm)	Relative Error (%)
*L*_1_	7.00	6.99 ± 0.01	0.14
*L*_2_	15.00	15.05 ± 0.01	0.33
*R*_3_	20.00	20.03 ± 0.01	0.15
*L*_4_	8.00	7.93 ± 0.01	0.88

**Table 2:** Key dimensions of the microchannels.

Key Dimension	Design Value (mm)	Measured Value (mm)	Relative Error (%)
*L*_6_	0.80	0.82 ± 0.01	2.50
*L_7_*	0.80	0.95 ± 0.03	18.75
*L*_8_	0.50	0.51 ± 0.01	2.00
*L*_9_	0.50	0.60 ± 0.02	20.00

## Data Availability

The datasets generated and supporting the findings of this article are obtainable from the corresponding author (zhaody@dlut.edu.cn) upon reasonable request.
